# A Case of Recurrent Breast Cancer with Solitary Metastasis to the Urinary Bladder

**DOI:** 10.1155/2014/931546

**Published:** 2014-03-04

**Authors:** Carsten Nieder, Adam Pawinski

**Affiliations:** ^1^Department of Oncology and Palliative Medicine, Nordland Hospital, P.O. Box 1480, 8092 Bodø, Norway; ^2^Institute of Clinical Medicine, Faculty of Health Sciences, University of Tromsø, 9037 Tromsø, Norway

## Abstract

Elderly patients with breast cancer often present with symptomatic, locoregionally advanced rather than screening-detected disease, thereby increasing the risk of metastatic recurrence during their remaining life time. Typical sites of metastases include lungs, bones, liver, and brain. Here we present a patient who developed a solitary urinary bladder metastasis five years after primary diagnosis of stage T4 N0 estrogen receptor-positive lobular carcinoma, while on continued adjuvant endocrine treatment (91 years of age). Anemia and increased serum creatinine resulting from hydronephrosis led to diagnosis of metastatic disease, which was confirmed by transurethral resection. The patient responded clinically to palliative radiotherapy and a different type of endocrine therapy. One year after diagnosis of metastatic disease, she died without signs of cancer progression.

## 1. Introduction

Elderly patients with breast cancer often present with locally advanced and/or symptomatic disease because screening programs typically focus on younger women [1]. It has long been recognized that patients diagnosed with locally advanced disease also face a higher risk of metastatic recurrence during the course of disease [2]. Autopsy series from the last century showed that most distant metastases are located in the lymph nodes, lungs, pleura, bones, adrenal glands, liver, and brain [3]. However, other organs such as pituitary gland, kidneys, uterus, and thyroid gland might also be affected. Compared to these, the urinary bladder is a very rare site of distant relapse [4]. It is even more unusual that this type of metastatic spread is limited to only one organ. Here we report the clinical course of a patient with solitary metastasis to the urinary bladder.

## 2. Case Report

In November 2007, an 86-year-old Caucasian female was diagnosed with left-sided clinical stage T4b N0 breast cancer, involving the whole breast and infiltrating the skin. She received primary endocrine treatment with letrozole. Her tumor responded well and in April 2008, mastectomy was performed. Histology showed lobular carcinoma grade I with 100% estrogen receptor (ER) positivity and negative progesterone receptor (PR) and Her-2 expression. Postoperatively, she continued on letrozole.

**Figure 1:**
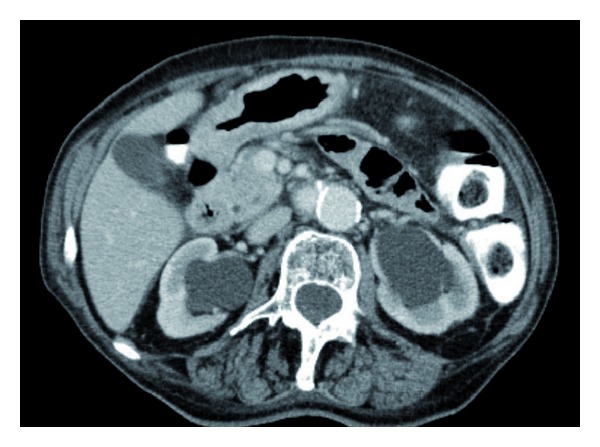
Axial computed tomography scan of the abdomen showing bilateral hydronephrosis without enlarged retroperitoneal lymph nodes.

**Figure 2:**
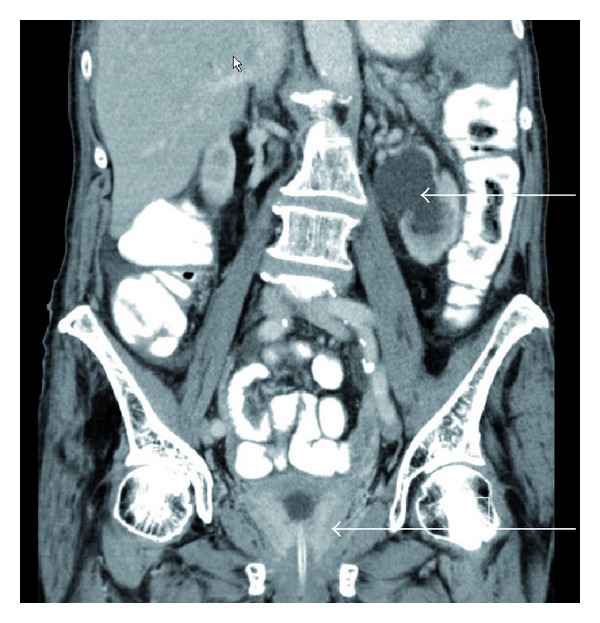
Computed tomography scan of the abdomen and pelvis showing both hydronephrosis (upper arrow) and thickening of the bladder wall (lower arrow), bladder catheter in situ.

**Figure 3:**
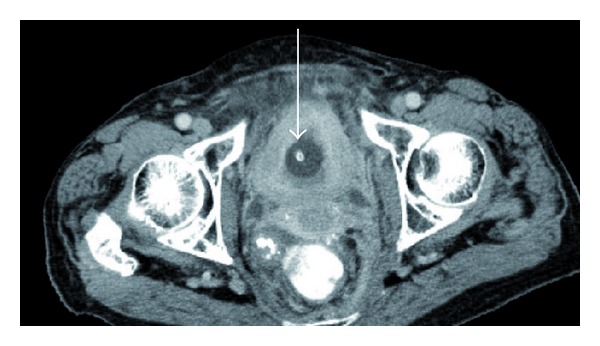
Axial computed tomography scan of the pelvis showing a diffuse infiltration of the bladder wall, catheter in situ (arrow).

In November 2012, she was hospitalized because of anemia (serum hemoglobin 6.9 g/dL) and reduced kidney function (serum creatinine 143 *μ*mol/L). Ultrasound examination revealed bilateral hydronephrosis, and computed tomography (CT) of the chest, abdomen, and pelvis confirmed this finding (Figure 1). Furthermore, a diffuse tumor infiltration in the urinary bladder outlet was seen (Figures 2 and 3, thickened bladder wall). No lymph node or distant metastases were found. No locoregional relapse was detected either. Cystoscopy revealed a bleeding tumor in the lower parts of the bladder mucosa. Transurethral resection was performed and a bladder catheter was inserted. Afterwards, kidney function returned to normal. The patient also received red blood cell transfusions. Blood chemistry was unremarkable, except for elevated tumor markers (carcinoembryonic antigen 49 *μ*g/L and CA-125 378 ku/L). Histology showed metastasis from lobular breast cancer with ER positivity in 50% of the tumor cells (PR and Her-2 negative). The cells also stained strongly positive for gross cystic disease fluid protein 15 (GCDFP).

Systemic treatment was switched from letrozole to tamoxifen. In addition, the patient received palliative 3D conformal radiotherapy to the urinary bladder (January 2013). Ten fractions of 3 Gy were given. Further red blood cell transfusions were necessary during the next few months, but not from June onwards, indicating delayed response to treatment. Serum creatinine remained stable at 90–110 *μ*mol/L. Given this symptomatic improvement and the patient's age of 91 years at that time, no further CT scans or tumor marker analyses were ordered. She died in a nursing home, 92 years old and without signs of cancer progression. Survival was six years from initial breast cancer diagnosis, and one year from diagnosis of metastatic disease.

## 3. Discussion

Compared to common sites of breast cancer metastases such as lungs, bones, and liver, spreading to a large number of organs including heart [5], pancreas [6], and gastrointestinal tract is rare [7]. This is also true for the urinary bladder. According to our PubMed search, an early case was reported in 1965 [8], followed by occasional publications and a summary in 2005 [9]. In a more recent series that also included few cases with other primary tumors, 90% of the patients presented with hematuria and/or obstructive urinary symptoms as well as bladder lesions in the area of trigone, posterior wall, and/or bladder neck [4]. Seven of the 11 patients had a known history of other metastases besides the bladder. Most of the patients (4/7, 57%) died within one year after diagnosis of bladder metastasis. The few reported cases typically harbored metastases to other organs, but Lin and Chen also reported a patient whose disease recurrence was limited to the bladder [10]. This patient relapsed three years after initial diagnosis of ER negative Her-2 positive ductal carcinoma. The metastasis was GCDFP positive, as in our case. Primary systemic therapy was given and the patient was alive at the time of writing (two years). Few reports concerned patients with lobular breast cancer. The patient described by Shah et al. had metastatic disease already at first diagnosis, with hydronephrosis as initial sign of malignancy [11]. Despite chemotherapy, she survived for only six months. A different patient with lobular cancer developed bladder metastasis six years after initial cancer diagnosis, with simultaneous local relapse [12]. She also received chemotherapy, and unfortunately survival was not reported. Overall, it seems that different biological types of breast cancer have the ability to metastasize to the urinary bladder, after variable time intervals. Especially if no other metastases are known, diagnosis might be challenging. Histological confirmation appears necessary if one wants to rule out primary bladder cancer, which typically originates from urothelial cells, but other variants such as squamous cell and small cell cancers can also be found [13]. Systemic treatment is not different from that of metastatic breast cancer in general. However, local radiotherapy should be considered because of its ability to stop hematuria [14, 15] and provide local disease control.
